# The Relationship Between the miRNA Sequence and Disease May be Revealed by Focusing on Hydrogen Bonding Sites in RNA–RNA Interactions

**DOI:** 10.3390/cells8121615

**Published:** 2019-12-11

**Authors:** Tatsunori Osone, Naohiro Yoshida

**Affiliations:** 1 School of Materials and Chemical Technology, Tokyo Institute of Technology, Yokohama 226-8502, Japan; 2 Earth-Life Science Institute, Tokyo Institute of Technology, Tokyo 152-8550, Japan; yoshida.n.aa@m.titech.ac.jp

**Keywords:** microRNA, regression analysis, hydrogen bond, disease

## Abstract

MicroRNAs are important genes in biological processes. Although the function of microRNAs has been elucidated, the relationship between the sequence and the disease is not sufficiently clear. It is important to clarify the relationship between the sequence and the disease because it is possible to clarify the meaning of the microRNA genetic code consisting of four nucleobases. Since seed theory is based on sequences, its development can be expected to reveal the meaning of microRNA sequences. However, this method has many false positives and false negatives. On the other hand, disease-related microRNA searches using network analysis are not based on sequences, so it is difficult to clarify the relationship between sequences and diseases. Therefore, RNA–RNA interactions which are caused by hydrogen bonding were focused on. As a result, it was clarified that sequences and diseases were highly correlated by calculating the electric field in microRNA which is considered as the torus. It was also suggested that four diseases with different major classifications can be distinguished. Conventionally, RNA was interpreted as a one-dimensional array of four nucleobases, but a new approach to RNA from this study can be expected to provide a new perspective on RNA-RNA interactions.

## 1. Introduction

According to the World Health Organization, the leading causes of death worldwide are stroke (ST), chronic obstructive pulmonary disease (COPD), Alzheimer’s disease (AD), and tuberculosis (TB) [1]. ST has been shown to have a higher incidence in developing countries than in developed countries. Of the various types of ST, 85% are ischemic. It has also been suggested that both genetic and environmental factors are involved in pathophysiology [2]. COPD is caused by an inflammatory response in the lungs to harmful gas or particles. COPD is progressive and often coexists with other diseases. The most common risk factor is smoking [3]. AD, the most common cause of dementia, is a progressive neurodegenerative disease that develops due to neuronal death and loss or atrophy of synaptic functions [4]. TB is an infectious disease caused by *Mycobacterium tuberculosis* that causes death beyond AIDS due to HIV. Currently, it is estimated that one-third of the world’s population is potentially infected. However, it affects about 5–10% of infected people [5].

MicroRNA (miRNA) is attracting attention as a treatment method and biomarker for these diseases [6]. miRNA is a class of small non-coding RNA, containing around 22 nucleotides. miRNA mainly controls the turnover and translation of target mRNA by binding the 3’ untranslated region of mRNA as a target site [7]. Currently, seed theory is the mainstream in binding miRNA targets [8]. Seed theory states that the 2nd to 8th bases of miRNA bind complementarily to the target site. Various target prediction programs have been developed based on this theory, but it is known that they often have high false positive rates or false negative rates [9]. miRNAs are related to various biological processes because of their functions, and their relation to diseases is well studied [10,11]. In order to clarify the control mechanism of biological process by miRNA, a new theory for the miRNA mechanism may be necessary. For this reason, several studies have recently been conducted to clarify the relationship between diseases and miRNAs using methods that are not based on seed theory. Zou et al. combined the miRNA–miRNA network, miRNA–disease network, and disease–disease network, and performed network analysis by machine learning [12]. Luo et al., Zou et al., Lan et al., and Fu et al. applied network analysis by machine learning to miRNA–disease networks [9,13,14,15]. Ding et al. focused on miRNA clusters/family and secondary structure [16], and Zheng et al. focused on miRNA’s functional similarity and semantic similarity to diseases [17]. These methods can be expected to enable prediction of disease-related miRNA candidates that have not yet been found. For example, *hsa –let –7d*, *hsa –mir –18a*, *hsa –mir –145*, *hsa –mir –106b*, *hsa –let –7e*, *hsa –let –7b*, *hsa –mir –19a*, and *hsa –mir –125a* may be related to breast cancer [12].

Seed theory may be superior in terms of the mechanism by which miRNA suppresses translation of mRNA and the prediction of miRNA-mRNA networks. In addition, from the viewpoint of predicting disease-related miRNA candidates that have not yet been found, the above-described approach using machine learning is considered to be excellent. However, neither method may be suitable in terms of decoding the miRNA gene. This is because seed theory focuses on sequence specificity when miRNA is viewed as a translation inhibitor of mRNA, and machine learning approaches are not based on sequences. Decoding miRNA genes not only reveals the mechanism of miRNA-mRNA interactions and miRNA-non-coding RNA interactions, but may also reveal the role of short RNAs in primitive life, such as in its birth and evolution.

In order to clarify the relationship between biological phenomena and miRNA sequences, the concept of black box modeling in system identification in the engineering field was used. In black box modeling, an internal system is regarded as a black box, and system identification is performed using only Input and Output. In this study, Input was designated as miRNA sequence and Output as disease. The RNA-RNA interaction was the focus of attention when quantifying miRNA sequences. The purpose of this study was to verify whether this new approach reveals the relationship between miRNA and ST, COPD, AD, and TB.

## 2. Materials and Methods

### 2.1. Datasets

Research papers on miRNA in AD, COPD, ST, and TB using patient tissues (33, 23, 31, and 21 reports, respectively) were selected. (Table S1). When searching, “microrna Disease Name” was used as a keyword. From those papers, the name of miRNAs showing the expression level different from the control and these expression levels were extracted. If the expression level could not be obtained, the value of fold-change (FC) or log_2_FC was obtained. If no value was obtained in the text or in the table, the value was obtained from the graph using WebPlotDigitizer (https://apps.automeris.io/wpd/). The sequence information of miRNA was obtained from miRbase Release 22.1 (http://www.mirbase.org/).

### 2.2. Convert Nucleobase to Vector

Adenine (A), guanine (G), cytosine (C), and uracil (U) modelled using Avogadro (©2018 Avogadro Chemistry, v1.2.0) have undergone 25 structural optimizations. After that, it was output as a GAMESS Input file, with the calculated as single point energy, using B3LYP/6–31G (d) as the method, and water as the solvent.

**Figure 1:**
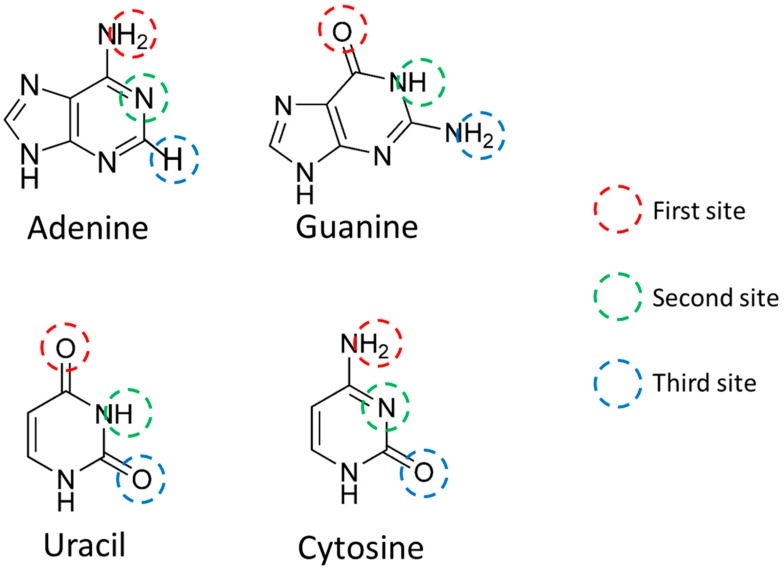
Site where nucleobase hydrogen bonds are located. Each of the three sites was designated as the First site (red circle), the Second site (green circle), and the Third site (blue circle). In addition, third site includes Adenine C2 hydrogen that is not used for RNA–RNA interactions.

The output file was calculated with Firefly QC package [18], which is partially based on the GAMESS (US) [19] source code, via MoCalc2012. Among the calculation results, MULLIKEN ATOMIC CHARGES of Atoms Used for Hydrogen Bonding (AUHB) of nucleic acids (Figure 1) was used as a vector indicating each nucleobase. When this vector is $\vec{B}$ and the element at the i−th site is $e_{i}$, the vector indicating the nucleobase is represented by the following formula (Equation (1)):

(1) $$\vec{B}=\left(e_{1},\;e_{2},\;e_{3}\right)$$

### 2.3. Scoring of miRNAs

miRNA scoring was performed by considering miRNA as a ternary vector with quantified nucleobases (Note: not a three-dimensional vector). Here, the normal multidimensional vector DV is represented as $\vec{\mathrm{DV}}$, and the multi-element vector EV is represented as $\vec{\vec{\mathrm{EV}}}$. At this time, assuming that the ternary vector in miRNA is $\vec{\vec{\mathrm{miR}}}$, the sequence length is n, and the element at the i–th site of the j–th nucleobases is $e_{i_{j}}$, the vector indicating the nucleobase is expressed by the following formula (Equation (2)):

(2) $$\vec{\vec{\mathrm{miR}}}=\left(\left(e_{1_{1}},\;e_{1_{2}},\;e_{1_{3}},\ldots ,\;e_{1_{n}}\right),\;\left(e_{2_{1}},\;e_{2_{2}},\;e_{2_{3}},\ldots ,\;e_{2_{n}}\right),\;\left(e_{3_{1}},\;e_{3_{2}},\;e_{3_{3}},\ldots ,\;e_{3_{n}}\right)\right)$$

In applying to disease, it was verified by four methods.

The first method was based on a mathematical method and scored with element vector size (VS). $\vec{\mathrm{VS}}$ is expressed by the following equation from Equation (2) (Equation (3)):

(3) $$\vec{\mathrm{VS}}=\left(\sqrt{e_{1_{1}}^{2}+e_{1_{2}}^{2}+\ldots +e_{1_{n}}^{2}},\;\sqrt{e_{2_{1}}^{2}+e_{2_{2}}^{2}+\ldots +e_{2_{n}}^{2}},\;\sqrt{e_{3_{1}}^{2}+e_{3_{2}}^{2}+\ldots +e_{3_{n}}^{2}}\right)$$

The second method is based on the electromagnetic method and the sum of electric charges (Sum) was used (Equation (4)):

(4) $$\vec{\mathrm{Sum}}=\left(e_{1_{1}}+e_{1_{2}}+\ldots +e_{1_{n}},\;e_{2_{1}}+e_{2_{2}}+\ldots +e_{2_{n}},\;e_{3_{1}}+e_{3_{2}}+\ldots +e_{3_{n}}\right)$$

The third method is based on the electromagnetic method and used the centre of the electric field vector (EV_C) of a miRNA structure. When the coordinates of the element at the i–th site of the j–th nucleobases are $\left(x_{i_{j}},\;y_{i}{}_{j},\;z_{i}{}_{j}\right)$, $\vec{\mathrm{EV}\_C}$ is expressed by the following formula (Equation (5)). The vector element of EV_C is the x, y, and z component of the electric field vector, unlike the above two methods:

(5) $$\vec{\mathrm{EV}\_C}\propto \left(\begin{array}{c} \sum_{i=1}^{3}\sum_{j=1}^{n}\frac{e_{i}{}_{j}\left(x_{k}-x_{i_{j}}\right)}{\left({\left(x_{k}-x_{i}{}_{j}\right)}^{2}+{\left(y_{l}-y_{i}{}_{j}\right)}^{2}+{\left(z_{m}-z_{i}{}_{j}\right)}^{2}\right){\;}^{\frac{3}{2}}}\\ \begin{array}{c} ,\;\sum_{i=1}^{3}\sum_{j=1}^{n}\frac{e_{i}{}_{j}\left(y_{k}-y_{i_{j}}\right)}{\left({\left(x_{k}-x_{i}{}_{j}\right)}^{2}+{\left(y_{l}-y_{i}{}_{j}\right)}^{2}+{\left(z_{m}-z_{i}{}_{j}\right)}^{2}\right){\;}^{\frac{3}{2}}} \end{array}\\ ,\sum_{i=1}^{3}\sum_{j=1}^{n}\frac{e_{i}{}_{j}\left(z_{k}-z_{i_{j}}\right)}{\left({\left(x_{k}-x_{i}{}_{j}\right)}^{2}+{\left(y_{l}-y_{i}{}_{j}\right)}^{2}+{\left(z_{m}-z_{i}{}_{j}\right)}^{2}\right){\;}^{\frac{3}{2}}} \end{array}\right)$$

The fourth method is based on the electromagnetic method, and was the sum of the electric field vectors (EV_S) on the lattice points in the miRNA structure. The lattice points were included in the structure, and the maximum distance of each axis of *x*, *y*, and *z* was divided into 11 equal parts. When the lattice point at the a–th position among the 11 divisions is ($x_{a},y_{a},z_{a}$), $\vec{\mathrm{EV}\_S}$ is expressed by the following expression (Equation (6)). Note that the vector elements of EV_S are the x, y, and z components of the electric field vector as in $\vec{\mathrm{EV}\_C}$:

(6) $$\vec{EV\_S}\propto \left(\begin{array}{c} \sum_{k=-5}^{5}\sum_{l=-5}^{5}\sum_{m=-5}^{5}\sum_{i=1}^{3}\sum_{j=1}^{n}\frac{e_{i}{}_{j}\left(x_{k}-x_{i_{j}}\right)}{\left({\left(x_{k}-x_{i}{}_{j}\right)}^{2}+{\left(y_{l}-y_{i}{}_{j}\right)}^{2}+{\left(z_{m}-z_{i}{}_{j}\right)}^{2}\right){\;}^{\frac{3}{2}}}\\ ,\;\sum_{k=-5}^{5}\sum_{l=-5}^{5}\sum_{m=-5}^{5}\sum_{i=1}^{3}\sum_{j=1}^{n}\frac{e_{i}{}_{j}\left(y_{k}-y_{i_{j}}\right)}{\left({\left(x_{k}-x_{i}{}_{j}\right)}^{2}+{\left(y_{l}-y_{i}{}_{j}\right)}^{2}+{\left(z_{m}-z_{i}{}_{j}\right)}^{2}\right){\;}^{\frac{3}{2}}}\\ ,\;\sum_{k=-5}^{5}\sum_{l=-5}^{5}\sum_{m=-5}^{5}\sum_{i=1}^{3}\sum_{j=1}^{n}\frac{e_{i}{}_{j}\left(z_{k}-z_{i_{j}}\right)}{\left({\left(x_{k}-x_{i}{}_{j}\right)}^{2}+{\left(y_{l}-y_{i}{}_{j}\right)}^{2}+{\left(z_{m}-z_{i}{}_{j}\right)}^{2}\right){\;}^{\frac{3}{2}}} \end{array}\right)$$

**Figure 2:**
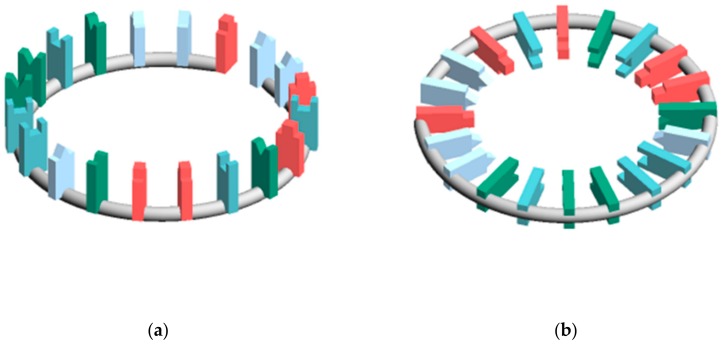

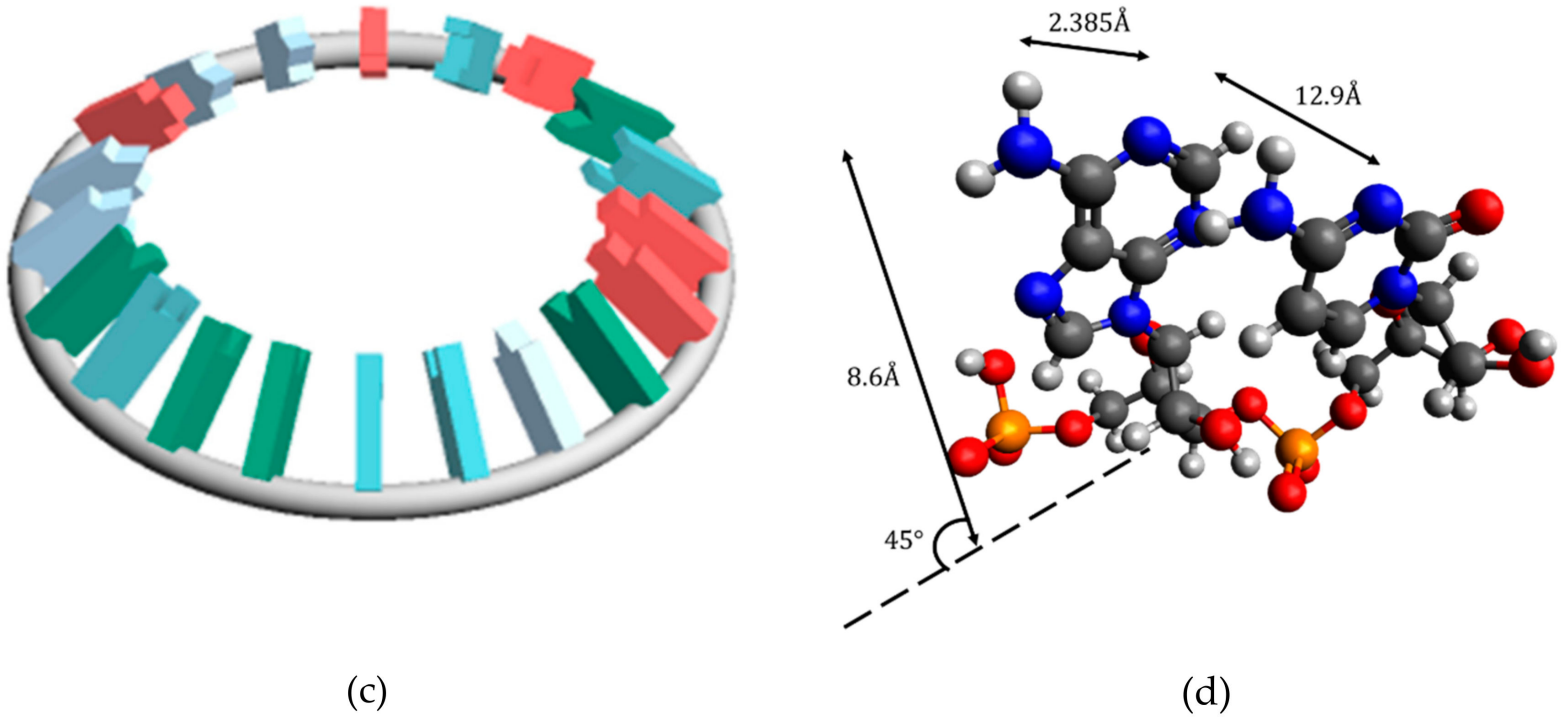
Placement of nucleobases relative to the circle (**a**) Structure that is most susceptible to hydrogen bonding with other RNA (Structure A) (**b**) Structure that is most stable (Structure B) (**c**) Structure between A and B (Structure C) (**d**) Figure excerpted and enlarged from part of structure C.

In calculating $\vec{\mathrm{EV}\_C}$ and $\vec{\mathrm{EV}\_S}$, the miRNA structure was regarded as a torus. This is because the existence of circular RNA is known and the topologically toric body has a high degree of freedom. Three types of miRNA structures were defined (Figure 2). In the structure X, $\vec{\mathrm{EV}\_C}$ and $\vec{\mathrm{EV}\_S}$ is defined as $\vec{\mathrm{EV}\_C\_X}$ and $\vec{\mathrm{EV}\_S\_X}$, respectively. In each structure, the distance between bases was 12.9 Å, the nucleobases length was 8.6 Å, and the distance between sites was 2.385 Å. In Structure C, the angle between the nucleobases and the surface formed by the torus was 45°.

### 2.4. Application to Diseases

The $\vec{\vec{\mathrm{miR}}}$ calculated by each method was used as the miRNA score ($\vec{\mathrm{miR}}$). When FC or log_2_FC are x and the score of the disease obtained therefrom is Score Multiplication Expression Level (SMEL), expressed by the following formula (Equation (7):

(7) $${\mathrm{SMEL}}_{\vec{\mathrm{miR}}}=\vec{\mathrm{miR}}x$$

## 3. Results

### 3.1. Evaluation of Each Scoring Method

The charge at each AUHB of each nucleobase was calculated by Firefly (Table 1).

For example, since the first site of A is an amino group, there are two hydrogen atoms (H) that form hydrogen bonds. Although different values were obtained in the calculation results, the average value was defined as the H charge in this study. In the Watson–Crick pair, A and U are hydrogen bonded only at the first site and the second site. This is because the distance between H present at the third site of A and the oxygen atom (O) present at the third site of U is large. However, U is known to form a wobble base pair with G. In the wobble base pairing, the second site of U and the first site of G, and the third site of U and the second site of G, are hydrogen bonded. Therefore, the third site of U was also used for scoring miRNA. Regarding the third site of A, it was used for scoring miRNA for the following two reasons: (1) since the three scoring methods except VS are based on electromagnetic methods and (2) to unify into the same ternary vector as the other three nucleobases.

The proportion of unique values in $\vec{\mathrm{miR}}$ of each method was verified. In addition, there are 24 duplications in the miRNA sequence and the total number of unique sequences is 2632 (Table 2).

EV_S_B and EV_S_C have a nearly 1: 1 relationship with the miRNA sequence. On the other hand, VS was the most duplicated. EV_C type and EV_S type differ greatly in the degree of overlap, and EV_C type had more overlap. In addition, the EV_C/S type had more overlap in structure A than in others.

Furthermore, single regression analysis was performed for each method (Table 3). The First element or the x component of $\vec{\mathrm{miR}}$ was taken as the *x*-axis, the Second element or the y component of $\vec{\mathrm{miR}}$ was taken as the *y*-axis, and the Third element or the z component of $\vec{\mathrm{miR}}$ was taken as the *z*-axis. Sum had the lowest correlation compared to other methods.

### 3.2. Single Regression Analysis for SMEL

**Figure 3:**
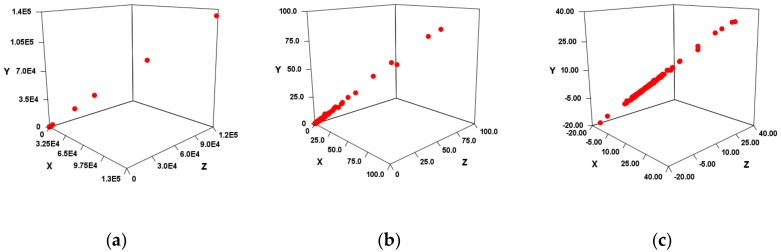
3D scatter plot of VS calculation results in AD. The *X*-axis was the First site score, the *Y*-axis was the Second site score, and the *Z*-axis was the Third site score. (**a**) Result when using fold change. (**b**) Figure enlarging the range of 0 to 200 in Figure 3a. (**c**) Result when using log_2_FC.

MiRNAs with significantly different expression levels compared to healthy controls were obtained from the paper data for four diseases (Table S1). If both healthy control and patient miRNA expression data were obtained, FC and log_2_FC values were calculated. When only the FC value was obtained, the log_2_FC value was calculated, and when only the log_2_FC value was obtained, the FC value was calculated. In each of the FC groups and log_2_FC groups for the miRNA group thus obtained, SMEL by each $\vec{\mathrm{miR}}$ was calculated. As an example, ${\mathrm{SMEL}}_{\vec{VS}}$ of AD is shown (Figure 3).

As a result, in both FC (Figure 3a) and log_2_FC (Figure 3b), a very high correlation was suggested for each combination of *x*-axis, *y*-axis, and *z*-axis. In other words, each site that is an independent variable is considered to have multicollinearity. However, each independent variable should not be synthesized because it depends on the hydrogen bonding site of the nucleobase. Therefore, instead of multiple regression analysis, single regression analysis was performed with all independent variable combinations (Table 4). As a result, both FC and log_2_FC were found to be highly correlated except for Sum, EV_C_B, and EV_C_C.

### 3.3. Validation of the Most Suitable Method

Since SMEL is the product of $\vec{\mathrm{miR}}$ and FC or log_2_FC, the intercept is zero. Therefore, the regression line for each disease is non-parallel. Thus, whether there was a significant difference in the regression line of SMEL for each disease was examined by standard deviation instead of covariance analysis. In VS, it was shown whether there was a significant difference in the inclination of the *x*-axis and *y*-axis (Table 5).

When calculated by FC, it was shown that there was a significant difference in slope in all combinations when ±2σ. On the other hand, in the case of log_2_FC, there was a significant difference in ST and other diseases, but there was no significant difference in other combinations. Based on Table 5, the case where there was a significant difference in all combinations of diseases was marked as ◯, and the case where there was a combination with no significant difference was marked as × (Table 6).

Then, there was an axis with a significant difference in FC, whereas there was no axis with a significant difference in log_2_FC. In the case of ±2σ, the number of ◯ exceeded 50% in EV_C type, EV_S_B, and EV_S_C. Since EV_C_A has only the *x* –axis and *y* –axis, the number of ◯ was interpreted as 100%. However, in the single regression analysis of each disease, EV_C_B and EV_C_C p > 0.1, and the correlation was low.

### 3.4. Reassessment Considering Outliers

Unlike log_2_FC, FC may have a very large value (Figure 3a). Therefore, when the FC values are sorted in descending order and a value that is twice as large, or larger, than the next smallest value appears, the larger value is regarded as an outlier.

Unlike the case where outliers were included, the same calculation was performed except without the outliers. However, ±3σ was all ×, so ±1σ and ±2σ were examined (Table 7). When the outliers were excluded, the only significant difference in ±2σ was in the *y* –*z* axis in EV_S_B. In addition, ±1σ in EV_S_B did not change from ±2σ.

## 4. Discussion

Since miRNA is deeply involved in various biological reactions including diseases, it is important to know the mechanism in detail in the treatment of diseases [20,21]. The mechanism by which miRNA suppresses translation has been clarified [22], but the meaning of the sequence when miRNA is regarded as a gene has not yet been clarified. The seed region is considered important in the seed theory, but the array outside the seed region has not been fully elucidated. Even in the seed region, the relationship with the disease is unclear in terms of the base sequence. Elucidating the relationship between miRNA sequences and diseases is the first step to deciphering miRNA genes. However, it seems that conventional methods are difficult in terms of decoding miRNA genes. Therefore, we focused on RNA–RNA interactions. Nucleobase hydrogen bonds are utilized when reacting with other nucleic acids. Therefore, the base sequence was digitized by using AUHB as the miRNA score, and the relationship with the disease was verified.

In order to quantify the miRNA sequence, the charge was calculated focusing on AUHB (Table 1). The H was a positive value, and O and nitrogen atoms were negative values. In the Watson–Crick pair, the third site of A does not bond with hydrogen, and in this charge calculation, only the charge of the third site of A was smaller in absolute value than other atoms. Therefore, this calculation result was interpreted as appropriate.

In this study, miRNA was considered a torus. There are two reasons for this. First of all, it is known that there are circular forms of various RNA types. For example, circular RNAs have been reported [23,24]. In addition, tRNA is clover-shaped and can be regarded as circular in terms of topology. Pre-mRNA [25] and phytopathogenic virus viroid [26] are also circular. Although we have not found any miRNA reports that are circular, miRNA secondary structure suggests a circular shape. For example, the secondary structure of mature miRNA can be predicted with CentroidFold [27]. When predicting the secondary structure of miRNA in CentroidFold, miRNA basically has a dumbbell shape, but some miRNAs become torus (e.g., hsa-miR-1-5p; inference engine: CONTRAfold; weight of base pairs: 2^2^). The dumbbell type is also topologically similar to a torus. Second, considering miRNA as a torus has two advantages. The first is that the clover type and dumbbell type can be considered topologically homologous, so the method of this study may be applicable to other types of RNA. Secondly, the readability of the miRNA genetic code sequence is high. In order to decipher the genetic code of miRNA sequences as the ultimate goal, this study aims to search for pre-data processing methods for machine learning. It is probably difficult to perform data preprocessing because the amount of information is too small for a one-dimensional array. On the other hand, there is too much information in an accurate secondary structure or tertiary structure. A torus can be viewed as an approximation of secondary structure.

Based on the calculation results, each $\vec{\mathrm{miR}}$ was calculated. In order to clarify the relationship between miRNA sequences and diseases, it is desirable that $\vec{\mathrm{miR}}$ and sequences have a 1: 1 relationship. Therefore, the ratio of unique values in each $\vec{\mathrm{miR}}$ was verified (Table 2). The EV_C type and EV_S type differ greatly in the degree of overlap, and the EV_C type had more overlap. This is thought to be because the EV_C type is a vector with only the centre, so the influence from the charge is small, whereas the EV_S type is the sum of the electric field vectors in the torus, so it is strongly influenced by the charge. In addition, the EV_C/S type had more overlap in structure A than in others. This is presumably because the z component of the electric field vector does not exist. Surprisingly, Sum had a low overlap rate compared to the EV_C type and EV_S_A. From the viewpoint of unique values, Sum, EV_S_B, and EV_S_C were considered suitable.

A single regression analysis was performed to examine the distribution trend of each $\vec{\mathrm{miR}}$ value (Table 3). Overall correlation was high except for Sum. $\vec{B}$ is a value uniquely determined by the nucleobase, but $\vec{B}$ itself did not show similarities in the values of each element except for the values of the second and third sites of G. Nevertheless, it was unexpected that the correlation was high. Note that the strong correlation in the order of VS, EV_S, EV_C, and Sum is thought to be due to the difference in strength affected by $\vec{B}$. From the viewpoint of correlation, VS and EV_S types were considered suitable.

Since the correlation was high in each $\vec{\mathrm{miR}}$, it was suggested that SMEL could also have a high correlation. When tested in four diseases, VS and EV_S types showed a high correlation in SMEL, and EV_C type and Sum showed low correlation (Figure 3, Table 4). As described above, since the SMEL intercept is 0, the regression line of each disease is non–parallel. If there is no relationship between the miRNA sequence and the disease, it should be almost the same as the slope in $\vec{\mathrm{miR}}$. However, there was actually a significant difference in the slope (Table 3 and Table 4). In other words, the high correlation of SMEL is thought to be due to the correlation between the disease and miRNA, although it is influenced by the high correlation of $\vec{\mathrm{miR}}$.

Therefore, it was verified whether the disease can be distinguished by SMEL (Table 5 and Table 6). As a result, EV_C_A, EV_S_B, or EV_S_C was considered a suitable method. However, there may be large outliers in the case of FC (Figure 3a,b). Since these are linear regressions, outliers can have a significant impact in the case of FC. Therefore, it was verified even when outliers were not taken into account (Table 7). Based on the results with and without outliers, the most suitable $\vec{\mathrm{miR}}$ was considered to be EV_S_B.

EV_S_B was suitable for unique values, correlations, and SMEL. The reason why EV_S_C, which showed the same tendency as EV_S_B, did not show a significant difference at 2σ of SMEL, seems to be because EV_S_B was closest to the possible structure.

**Figure 4:**
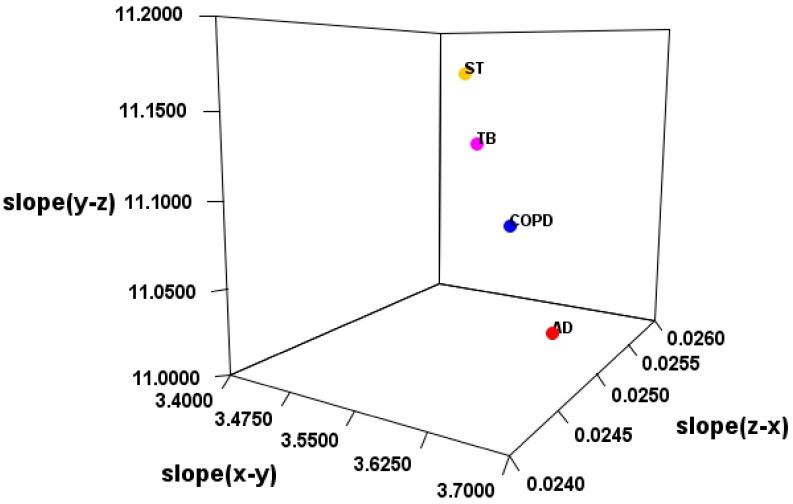
Slope of 3D regression line for each disease.

It was suggested that the classification of the disease is possible by the electric field vector made by AUHB (Figure 4), but there are three main problems in this result. The first is that the structure is simplified. In the case of EV_S_B, which seemed most suitable, the First to Third sites were placed perpendicular to the plane of the torus (Figure 2b). However, the actual nucleobase of miRNA is not vertical, but seems to be angled by the π–π interaction and the phosphate–sugar chain interaction. Therefore, it is necessary to perform calculations in consideration of the angle. The second is that only four types of disease are compared. Thus, in order to prove the hypothesis that “the relationship between the miRNA sequence and disease may be revealed by focusing on hydrogen bonding sites in RNA–RNA interaction”, more diseases must be targeted. Furthermore, in this study, comparisons were made between different diseases in major classifications, but it is also necessary to verify that diseases with the same large category, such as cancer types, can be distinguished. The third is interpretation of the score. Since EV_S_B is almost sequence-specific, the results of this study are considered to be one step closer to miRNA gene decoding. However, as mentioned in the second problem, there are still only few subjects tested in the experiment. Therefore, it is not possible at this time to interpret the score. In order to interpret the score, it is necessary to collect a lot of data and perform machine learning. Therefore, it is necessary to continue research in the future.

## 5. Conclusions

By considering RNA as a ternary vector, it was suggested that the sequence and its expression may be related to the disease. This study is a step toward clarifying the meaning of the sequence of miRNA genes by a different approach from the conventional “method of focusing on complementary sequences of miRNA” and “analysis of gene networks”. Until now, the gene has been interpreted as a one-dimensional sequence of A/U/G/C. From now on, it can be expected that a new viewpoint for RNA-RNA interaction will be obtained by looking at a ternary vector using AUHB.

## Figures and Tables

**Table 1:** Charge at the site of hydrogen bonding in base pair.

Nucleobase	First	Second	Third
Adenine	0.417	−0.514	0.228
Uracil	−0.506	0.408	−0.471
Guanine	−0.490	0.414	0.413
Cytosine	0.379	−0.558	−0.476

**Table 2:** Duplicate in each scoring method.

Methods	Total Number of Duplicate Values	Total Number of Unique Values	Percentage of Unique Values
VS	960	1696	64
Sum	454	2202	83
EV_C_A	832	1824	69
EV_C_B	706	1950	73
EV_C_C	715	1941	73
EV_S_A	615	2041	77
EV_S_B	78	2578	97
EV_S_C	65	2591	98
ref*	24	2632	99

* For reference, the duplicates of miRNA sequences were presented.

**Table 3:** 3D regression line of miRNA in each scoring method.

Methods	*x* − *y*				*y* − *z*				*z* − *x*			
	Slope	S.D.	*p*-Value	*r* ^2^	Slope	S.D.	*p*-Value	*r* ^2^	Slope	S.D.	*p*-Value	*r* ^2^
VS	1.04	0.01	2.E − 123	1.00	0.86	0.01	2.E − 126	1.00	1.10	0.01	4.E − 133	1.00
Sum	−0.65	0.07	1.E − 14	−0.67	0.45	0.09	1.E − 06	0.45	0.13	0.10	2.E − 01	0.11
EV_C_A	3.39	0.01	4.E − 150	1.00	NA	NA	NA	NA	NA	NA	NA	NA
EV_C_B	3.38	0.01	3.E − 148	1.00	0.02	0.00	9.E − 07	0.46	2.54	0.50	2.E − 06	0.45
EV_C_C	3.39	0.01	1.E − 149	1.00	0.02	0.00	1.E − 12	0.63	5.36	0.67	2.E − 12	0.62
EV_S_A	1.41	0.05	2.E − 48	0.93	NA	NA	NA	NA	NA	NA	NA	NA
EV_S_B	3.56	0.01	1.E − 149	1.00	11.08	0.01	7.E − 216	1.00	0.03	0.00	4.E − 153	1.00
EV_S_C	3.56	0.01	6.E − 150	1.00	11.12	0.03	2.E − 172	1.00	0.03	0.00	2.E − 141	1.00

NA: The item could not be calculated because there was no value.

**Table 4:** 3D regression line of AD in each scoring method.

Methods		*x* − *y*				*y* − *z*				*z* − *x*			
		Slope	S.D.	*p*-Value	*r^2^*	Slope	S.D.	*p*-Value	*r^2^*	Slope	S.D.	*p*-Value	*r^2^*
**log_2_FC**	VS	1.04	0.00	0.E + 00	1.00	0.85	0.00	0.E + 00	1.00	1.13	0.00	0.E + 00	1.00
	Sum	−0.25	0.04	2.E − 09	−0.30	0.12	0.08	1.E − 01	0.08	0.16	0.04	2.E − 05	0.21
	EV_C_A	3.31	0.01	0.E + 00	1.00	NA	NA	NA	NA	NA	NA	NA	NA
	EV_C_B	3.28	0.02	0.E + 00	1.00	0.02	0.00	7.E − 15	0.38	2.24	0.28	1.E − 14	0.38
	EV_C_C	3.29	0.01	0.E + 00	1.00	0.02	0.00	8.E − 33	0.56	4.77	0.36	7.E − 33	0.56
	EV_S_A	1.25	0.10	5.E − 33	0.56	NA	NA	NA	NA	NA	NA	NA	NA
	EV_S_B	3.52	0.01	0.E + 00	1.00	11.10	0.01	0.E + 00	1.00	0.03	0.00	0.E + 00	1.00
	EV_S_C	3.53	0.01	0.E + 00	1.00	11.10	0.01	0.E + 00	1.00	0.03	0.00	0.E + 00	1.00
**FC**	VS	1.03	0.00	0.E + 00	1.00	0.87	0.00	0.E + 00	1.00	1.11	0.00	0.E + 00	1.00
	Sum	0.06	0.02	5.E − 05	0.20	−0.64	0.14	4.E − 06	−0.24	0.88	0.04	2.E − 65	0.73
	EV_C_A	3.26	0.00	0.E + 00	1.00	NA	NA	NA	NA	NA	NA	NA	NA
	EV_C_B	3.26	0.00	0.E + 00	1.00	−0.01	0.00	1.E − 15	−0.39	−5.02	0.61	2.E − 15	−0.39
	EV_C_C	3.26	0.00	0.E + 00	1.00	0.00	0.00	6.E − 01	0.03	0.61	1.02	5.E − 01	0.04
	EV_S_A	1.39	0.00	0.E + 00	1.00	NA	NA	NA	NA	NA	NA	NA	NA
	EV_S_B	3.41	0.00	0.E + 00	1.00	11.18	0.00	0.E + 00	1.00	0.03	0.00	0.E + 00	1.00
	EV_S_C	3.43	0.00	0.E + 00	1.00	10.95	0.01	0.E + 00	1.00	0.03	0.00	0.E + 00	1.00

**Table 5:** Comparison of x − y slope of 3D regression line in VS.

Expression ratio		2σ			3σ		
		COPD	ST	TB	COPD	ST	TB
**log_2_FC**	AD	×	◯	×	×	◯	×
	COPD		◯	×		◯	×
	ST			◯			◯
**FC**	AD	◯	◯	◯	×	◯	×
	COPD		◯	◯		◯	×
	ST			◯			◯

**Table 6:** Comparison of slope of the 3D regression line.

Methods		2σ			3σ		
		x − y	y − z	z − x	x − y	y − z	z − x
**log_2_FC**	VS	×	×	×	×	×	×
	Sum	×	×	×	×	×	×
	EV_C_A	×	NA	NA	×	NA	NA
	EV_C_B	×	×	×	×	×	×
	EV_C_C	×	×	×	×	×	×
	EV_S_A	×	NA	NA	×	NA	NA
	EV_S_B	×	×	×	×	×	×
	EV_S_C	×	×	×	×	×	×
**FC**	VS	◯	×	×	×	×	×
	Sum	×	×	◯	×	×	×
	EV_C_A	◯	NA	NA	◯	NA	NA
	EV_C_B	◯	×	◯	◯	×	◯
	EV_C_C	◯	◯	×	◯	×	×
	EV_S_A	×	NA	NA	×	NA	NA
	EV_S_B	◯	◯	◯	◯	×	◯
	EV_S_C	◯	×	◯	◯	×	×

**Table 7:** Comparison of slope of the 3D regression line without some outliers.

Methods		1σ			2σ		
		x − y	y − z	z − x	x − y	y − z	z − x
**log_2_FC**	VS	×	×	×	×	×	×
	Sum	×	×	×	×	×	×
	EV_C_A	◯	NA	NA	×	NA	NA
	EV_C_B	×	×	×	×	×	×
	EV_C_C	◯	×	×	×	×	×
	EV_S_A	×	NA	NA	×	NA	NA
	EV_S_B	×	×	×	×	×	×
	EV_S_C	×	×	×	×	×	×
**FC**	VS	×	◯	×	×	×	×
	Sum	×	◯	×	×	×	×
	EV_C_A	×	NA	NA	×	NA	NA
	EV_C_B	×	◯	◯	×	×	×
	EV_C_C	×	×	×	×	×	×
	EV_S_A	◯	NA	NA	×	NA	NA
	EV_S_B	×	◯	×	×	◯	×
	EV_S_C	×	◯	◯	×	×	×

## Supplementary Materials

The following are available online at https://www.mdpi.com/2073-4409/8/12/1615/s1, Table S1: reference for dataset of each disease.
